# Echoes of the Month

**Published:** 1923-05

**Authors:** 


### ECHOES OF THE MONTH.

### PREVENTING MENTAL DISEASES.

A joint Board of Research for mental disease was recently formed in connection with the city and the University of Birmingham. The chairman is Sir Gilbert Barling, Bart. (Vice-Chancellor of the University of Birmingham), and the hon. director, Sir Frederick W. Mott. The co-operation of mental hospital authorities in the Midlands is sought, and a circular has been issued explaining the scheme. Therein it is pointed out that the Board has been formed for the purpose of promoting research into mental disease, with the objects of assisting prevention, and encouraging the more effective curative treatment, of such disease. At present the Board consists of an equal number of representatives of the Birmingham University and the City Asylums Committee, but the co-operation of governing bodies of mental institutions outside the City will be welcomed. Certain funds are being provided for the inauguration of the work, and temporary laboratory accommodation has been arranged in connection with one of the city mental hospitals at Hollymoor, near Birmingham. It is understood, however, that as soon as possible the Birmingham University will offer the necessary laboratory accommodation, so that the work may be in direct association with the general pathological work of the University.

### THE OLDEST ENGLISH MEDICAL SOCIETY.

Lord Dawson of Penn, as President of the Medical Society of London, is to take the chair when the Society meets to celebrate its 150th anniversary, and the Prince of Wales has promised to be present. This is the oldest medical society in England, and has had an uninterrupted career of prosperity since it was founded by Dr. John Lettsom in 1773, and first met at his own house in Sambrook Court, Basinghall Street. Originally the society was restricted to a membership of 90—30 physicians, 30 surgeons and 30 apothecaries, the last group representing the general practitioners of the time. Dr. Lettsom had other claims to fame than the founding of the Society, for he was a man of wide interests and something of a benefactor, devoting much of his time to problems of sanitation, the condition of the poor, and the formation of general dispensaries and hospitals. It was Dr. Lettsom also who urged and advanced the need of his profession for a medical library, and that established by the Society in London is one of the best of its kind, containing some very valuable works of the fifteenth and early sixteenth centuries.

### TYPHUS INCREASE IN RUSSIA.

The sixth number of the "Epidemiological Intelligence Bulletin" issued by the Health Section of the League of Nations Secretariat shows that the incidence of typhus and relapsing fever was fully twice as great in 1922 as in 1921 in Russia, another extremely high epidemic wave having occurred in 1921-22. This wave was quite double the 1920-21 wave, but was less than half as great as the second wave of 1919-20, judging from the number of cases reported. In Poland no improvement in 1922 over 1921 was noted for typhus, and the prevalence of relapsing fever has greatly increased. In Latvia there was apparently an increase of typhus, but a decrease of relapsing fever.

### LOW DEATH RATE OF AMERICA AND CANADA.

The death rate for 1922 in the United States and Canada was 8·8 per 1,000—the lowest, save one, ever recorded, according to statistics just compiled by the Metropolitan Life Insurance Company. This record is based on the mortality report of millions of industrial policy holders or over one-eighth of the population of the United States and Canada. The facts are considered to afford the earliest taustworthy index of general health conditions in the two countries during the year just closed. For the last six months of the year 1922, the record was the best ever shown for any half-year in history covered by policy-holder statistics. But considering the whole year, the record for the previous year, 1921, shows a death-rate slightly lower, not quite 1 per cent. less. Had it not been for the outbreak of influenza epidemic in the early months of 1922, the year's health record would have been better than the minimum established in 1921. The 1922 death-rate for tuberculosis, 113·4 per 100,000, was the lowest ever recorded. Since 1911 the tuberculosis death-rate has been reduced nearly one-half. The year 1922 recorded the lowest typhoid fever death rate in the history of the insured. The figure was 5·6 per 100,000, a reduction of one-sixth from the rate from 1921 and of nearly three-fourths from the figure recorded in 1911.

### FAMILY LIFE FOR THE LUNATIC.

The "Manchester Guardian" gives an account of the small Belgian town of Gheel, where, from the seventeenth century, it has been customary for the inhabitants to share their dwellings with those of unsound mind. The townsfolk are proud of the fine official title of their town: "State Colony for the Familial Treatment of Mental Affection." For many centuries the tomb of St. Dymphre was the object of a pilgrimage, especially for those of unsound mind, and the population of Gheel grew accustomed to receiving as lodgers pilgrims who were more or less deranged. From 1852 the institution became official. Experience has abundantly demonstrated that even lunatics who are dangerous in their own family surroundings become absolutely calm and obedient in the colony—such is the influence of the change of scene and the gentle methods used by the inhabitants of Gheel in their dealings with the patients. On arrival the patient is placed in observation in the central asylum, where he remains long enough to allow of the study of the particular character of his ailment. The choice of a foster-family for the patient is a most important question, and sometimes presents delicate difficulties. It is generally sought so to arrange that the patient may continue his habitual conditions of life. Artisans live with families of the same trade, those who come from the larger towns are placed with middle-class families in the centre of the colony, while peasants are lodged with the farmers. Statistics show that 25 per cent. are cured or at least considerably improved and able to return to their families.

### POISON FROM GLASS BOTTLES.

That a dry substance can absorb lead and arsenic from a glass bottle has been successfully put forward as a defence by Messrs. Boots, the chemists, at the Birmingham Police-court. Messrs. Boots were summoned for selling at one of their shops a quarter of a pound of potassium carbonate not of the quality demanded which contained about 80 parts of lead per 1,000,000 and about 10 parts of arsenic per 1,000,000. When the carbonate left Nottingham it was absolutely pure. Then it was put into a bottle. The only conclusion that could be come to was that the lead and arsenic were derived from the bottle. Experiments, said the defendants' counsel, had been made confirming this, which was a new discovery.

### THE "HEALTH GAME."

An effort is being made, under the auspices of the League of Red Cross Societies, to induce children to take an interest in their health. It is largely felt that the older methods of precept and compulsion are inadequate, and that appeal must be made to the child's own instincts. "The child," Dr. Holt, president of the Child Health Organisation of America, declared recently, "must be made to feel that health is a game, with rules like other games." In order to induce this method of thinking, fairy tales, adapted to health purposes, have been written; health clubs have been formed and distinctive badges and dresses designed.

### BYRON'S LAMENESS.

After more than 100 years, Dr. H. C. Cameron believes he has solved the mystery of Lord Byron's lameness. To this defect Byron was very sensitive; it indeed poisoned his whole life. The poet tells how once, when he was a child, his mother in one of her fits of fury called him a lame brat, and how he quickly retorted, "I was born so, mother." Dr. Cameron has been discussing the question at the Royal Society of Medicine, and the "British Medical Journal" reports what he believes is the solution of the lameness. During his lifetime it was generally believed that the poet was club-footed, but in which foot, or whether it was in both feet, was never known. His boxing instructor, "Gentleman" Jackson, the pugilist, thought it was the left. His mother stated definitely that it was the right foot. Dr. Cameron points out that his lameness did not prevent Byron from playing cricket for Harrow, and in his famous swim across the Hellespont he was in the water more than an hour. The lasts on which his boots were made, and which were discovered after long search in 1897, were shown to the doctors, and they confirm the belief that the poet did not suffer from club-foot. After careful consideration, Dr. Cameron believes Byron suffered from "Little's disease," an affection in the lower limbs caused by an injury to the brain at birth, and first described by Little, the great orthopædic surgeon.

### GREEN STRYCHNINE.

After a series of experiments carried out by chemists and the makers of British dyes, the Pharmaceutical Society have recommended brilliant green to the Privy Council as a colouring for strychnine, and the Privy Council have now asked the General Medical Council for their opinion on the proposal. The final choice lay between green and methyl violet, which was discarded as being less distinctive. Further tests are now in progress with a view to selecting suitable dyes for sheep dip and weed killer, both of which contain arsenic.

### SLEEPING SICKNESS IN AMERICA.

"The U.S. Public Health Service has no statistics in regard to the prevalence of *encephalitis lethargica*, popularly known as sleeping sickness, that are sufficiently reliable and complete to warrant a statement as to the extent of the disease throughout the United States," says Surgeon-General H. S. Cumming. "The disease is 'reportable' by physicians in comparatively few States, and in the larger part of the country the only data available are based on newspaper reports. Moreover, the disease is rather easy to confuse with some other diseases, and its prevalence is therefore likely to be unduly magnified. Thus, in an investigation made by Dr. H. F. Smith, of the Public Health Service, of the 1918-19 epidemic, 22 per cent. of the supposed cases had to be excluded as being really cerebro-spinal meningitis, cerebral syphilis, brain abscess, tuberculous meningitis, epilepsy, poliomyelitis, hysteria, or acute alcoholism. The disease appears to be only difficultly communicable. Not a single secondary case is known to have occurred in the immediate families of the patients reported in 1918-19, although some 900 persons were exposed. The mortality is rather high. Of the 159 cases studied by Smith death resulted in 46, or 29 per cent. The appearance of encephalitis in epidemic form has, except for one epidemic reported from Austria, always been preceded by an epidemic of influenza."

### ROCKEFELLER FOUNDATION AND IMPERIAL HYGIENE.

In connection with the recent offer by the Rockefeller Foundation to establish an Imperial School of Hygiene in London, it is understood that the trustees are prepared to give additional evidence of their liberality on this head. In order to secure the appointment of a director of the schools at the earliest moment they will provide £4,000 a year towards his expenses and his salary. He would thus be ready to supervise the building and equipment of the school, a site for which has been acquired in Bloomsbury, close to that tentatively apportioned to the new London University, and it is hoped that the Imperial School of Hygiene will be completed within two years.

### THE TOLL OF NYSTAGMUS.

One of the penalties of the miner's calling is nystagmus. The theory which has been generally accepted in recent years is that the primary cause is deficient lighting in the pit. Now, however, Dr. Frederick Robson has advanced the theory that nystagmus is contracted independently of bad illumination, and has a direct relation to the percentage of volatile matter in the coal of a particular district. He has contrasted the soft, bituminous coal of Monmouth with the hard varieties found in Glamorganshire, pointing out that nystagmus was twice as common in the former as in the latter district. Interviewed on the subject, Mr. George A. Spencer, M.P., general secretary of the Notts Miners' Association, whilst not challengeing the possibility of there being some foundation for Dr. Robson's theory, urged that it was of secondary importance, and must not be allowed to interfere with the movement for lessening the incidence of the disease by improving the lighting in pits. "So far as one can judge in Notts," remarked Mr. Spencer, "Dr. Robson's theory is not supported by facts. We have as much nystagmus, if not more, in hard coal pits as in soft coal pits."

### A QUAKER GIFT TO FRANCE.

An article in the *Manchester Guardian* gives an interesting account of the work done in the big Maternity Hospitals at Châlons given by the Quakers of England and America to the Department of the Marne. "The Maternity" takes any woman of whatever class belonging to the Department. But this is by no means all its work. If you go into the left wing, with its high white rooms and blue decoration, you will see quasi-patients, born some of them as long as nine years ago. Their mother has come to have her baby. To save her all anxiety the youngest one of the family has come too. Perhaps, however, the most interesting thing about it all is that this hospital is one of the few foreign importations during the war which has really taken root and is more flourishing than ever. And it must be remembered that when nursing was taken out of the hands of the nuns there was really nobody properly qualified to take their place. French midwifery has an extremely high academic standard. Once, however, the baby has come, the nursing standard is low, and is often in the hands of the wrong class of woman. The immense innovation of the Châlons Hospital is not that it knows more than the average French maternity hospital, but that it has introduced the conception of nursing by the educated woman.

### HOW OUR FATHERS POISONED THEMSELVES.

An investigator's dip into the report made by the first committee of inquiry into food adulteration in Britain in 1855 reveals that "with the potted meats and fish, anchovies, red sauces or cayenne taken at breakfast, our fathers or grandfathers would consume more or less bole Armenian, Venetian red, red lead, or even bisulphuret of mercury or cinnabar. At dinner, with his curry or cayenne, he would run the chances of a second dose of lead or mercury; with the pickles, bottled fruits or vegetables, he would be nearly sure to have copper administered to him; while, if he partook of bonbons at dessert, there is no telling how many poisonous pigments he might consume."

### ITALY'S HEALTH RESORTS.

The Italian Government has decided to draw the attention of doctors to the claims of the Italian spas and their health-giving climates. A special tour has been arranged in co-operation with the Italian medical authorities, and the principal spas, including San Pellegrino, Agnaro, and Montecatini, will be visited. The tour will be conducted by the Italian State Railways, and the British party will leave London on May 29. They will be accompanied by Italian doctors who speak English fluently.

### DOLLS: WELL- AND ILL-FED.

The United States Public Health Service sent a novel exhibit to the International Centennial Exposition at Rio Janeiro. It consisted of two tables, at each of which two large dolls, supposed to be ten years of age, sit at dinner. The meals spread for them look equally appetising. One of them, however, includes no milk, no green vegetables, and no fresh fruits. The dolls at this table are a little better dressed than those at the other table; but their faces, though they still show evidences of former beauty, are colourless and their eyes are dull and are underlined heavily with dark half-moons. The dolls at the other table, on the other hand, are rosy cheeked, bright, and laughing. The effect both on parents and children was very striking. Day after day the space before the exhibit was crowded by mothers or teachers, pointing out the difference in the appearance of the "children" (dolls) who drank milk and ate proper food and those who did not.

